# Real-Time Lossless Compression Algorithm for Ultrasound Data Using BL Universal Code

**DOI:** 10.3390/s18103314

**Published:** 2018-10-02

**Authors:** Jung Hoon Kim, Sunmi Yeo, Jong Won Kim, Kyeongsoon Kim, Tai-Kyong Song, Changhan Yoon, Joohon Sung

**Affiliations:** 1 Graduate School of Public Health, Seoul National University, Seoul 08826, Korea; powerzenith@naver.com (J.H.K.), jsung@snu.ac.kr (J.S.); 2 Department of Electronic Engineering, Sogang University, Seoul 04107, Korea; yeosunmi@gmail.com; 3 Department of Healthcare IT, Inje University Kimhae, Gimhae 50834, Korea; jongwonkim@inje.ac.kr; 4 Department of Pharmaceutical Engineering, Inje University Kimhae, Gimhae 50834, Korea; kskim@inje.ac.kr; 5 Department of Biomedical Engineering, Inje University Kimhae, Gimhae 50834, Korea

**Keywords:** medical ultrasound, lossless compression, universal code, run-length encoding

## Abstract

Software-based ultrasound imaging systems provide high flexibility that allows easy and fast adoption of newly developed algorithms. However, the extremely high data rate required for data transfer from sensors (e.g., transducers) to the ultrasound imaging systems is a major bottleneck in the software-based architecture, especially in the context of real-time imaging. To overcome this limitation, in this paper, we present a Binary cLuster (BL) code, which yields an improved compression ratio compared to the exponential Golomb code. Owing to the real-time encoding/decoding features without overheads, the universal code is a good solution to reduce the data transfer rate for software-based ultrasound imaging. The performance of the proposed method was evaluated using *in vitro* and *in vivo* data sets. It was demonstrated that the BL-beta code has a good stable lossless compression performance of 20%~30% while requiring no auxiliary memory or storage.

## 1. Introduction

Conventional ultrasound imaging systems are based on special-purpose hardware such as application-specific integrated circuits (ASICs) and field-programmable gate arrays (FPGAs) [1,2,3]. These fixed-function chips can meet high data transfer rates and computation requirements for real-time ultrasound imaging. However, the low flexibility of hardware-based ultrasound imaging systems often requires considerable time and expense in deploying new features and applications. To mitigate the problem, many groups have developed several platforms of ultrasound imaging using programmable processors with high flexibility, which enable rapid prototyping and allow new applications to run on the same imaging platforms [4,5]. These ultrasound imaging systems generally employ a PC (personal computer) as the imaging host and graphic processing units (GPUs) or digital signal processors (DSPs) to support the higher computational complexity [6,7]. 

Although the high computational demands can be alleviated by using GPUs, the large amount of data transfer poses an additional problem that hinders the practical implementation of software-based architecture in ultrasound imaging systems. For example, ultrasound imaging systems with 128 channels, a sampling rate of 40 MHz, and a 12-bit analog-to-digital convertor’s (ADC) resolution require a data transfer rate of 10 GB/s. A commercially available ultrasound imaging system (i.e., Vantage, Verasonics Inc., Kirkland, WA, USA) can support a data transfer rate up to 6.6 GB/s via eight PCI express lanes [8]. Although the high data transfer rate can be supported by using multiple PCI express lanes, it would increase the cost and power consumption. Hence, this approach is only suitable for high-end ultrasound imaging systems. To adopt a software-based architecture in low-cost ultrasound imaging systems such as portable and hand-held ultrasound imaging systems, the data transfer should be achieved by using a popular interface such as a Universal Serial Bus (USB) [3,9].

Data compression has been investigated to reduce the amount of ultrasound data in software-based ultrasound imaging systems [10,11,12]. The MPEG (moving picture experts group) compression method was utilized to compress radio-frequency (RF) data. However, it requires high computation and memory resources for coding and decoding [11]. More recently, a lossless compression method for improving the performance of GPU-based beamformers was proposed [12]. However, this method needs additional memory (e.g., address memory) for decoding, and hence the compressed data can be larger than the original data because of the additional addresses. In addition, this method groups several data into a batch for compression; consequently, it does not permit independent data extraction. 

A universal code is a prefix code that maps integers onto binary codewords and is widely used in data compression [13]. The universal code is a lossless data compression method, and the binary codewords are uniquely decodable for random integers. The main advantage of the universal code is that it can be easily implemented through simple calculations. However, the compressed codes generated from an equivalent number of symbols are not optimal compared to ones generated by the dictionary method such as Huffman and Lempel–Ziv–Welch (LZW) codes [14]. To address this limitation, the authors have proposed a new universal code, called Binary-cLuster (BL) code, by modifying the conventional exponential Golomb code [15,16,17]. The proposed BL code shows better compression performances, especially for data sets with large integers. For example, the BL code encodes the integer 1,000,000 as a 26-bit binary number (i.e., 11100011110100001001000001), while Elias Gamma, Fibonacci and Elias Delta codes encode the same integer as a 39-bit, 30-bit and 28-bit binary number, respectively. In addition, the BL code can compress any sequence of integers in real time without any overhead data in encoding and decoding. 

In this paper, we propose a BL code-based ultrasound signal compression method for real-time software-based portable medical ultrasound imaging systems. It was shown that the BL code outperforms the exponential Golomb code regarding the compression ratio for both *in vitro* and *in vivo* ultrasound data sets. Compression ratios of the proposed method for various signals in the signal processing chain of the ultrasound imaging system, such as pre-beamformed, beamformed and baseband inphase/quadrature (*IQ*) data, were also evaluated.

## 2. Method

### 2.1. Prefix of BL Code

For random binary data, the datum in front of “10” is separated when “10” is encountered either from the top (left) to the bottom (right) direction or in the opposite direction. For example, “1011101010010101111101001…” can be separated into “1011/10/10/100/10/101111/10/1001.” These separated binary numbers are herein referred to as binary clusters. By doing so, binary clusters can be created from any binary data that starts with “10” [15]. Table 1 shows the first ten cluster patterns. Here, we refer the sequential index corresponding to each binary cluster as code-num. Note that the binary cluster must start with the seed binary cluster “10”. As the code-num increases, each bit of the binary cluster holding “0” is replaced by “1”, one at a time, from bottom to top. When all “0” bits are flipped to “1” except for that in the seed binary cluster “10”, the bit length of the binary cluster is increased by adding “0” on the right, and then every “1” is replaced by “0” except for the most significant bit (MSB). By doing so, each code-num is allocated to a distinct binary cluster and can be used for a universal code. 

The group index *K* in Table 1 represents the bit length (excluding the MSB) of the binary cluster for each code-num (*M*). For example, the binary cluster of *M* = 5 is “1001”; therefore, *K* is 3. We used this group index *K* for encoding and decoding. It can be found that *K* and *M* satisfy inequality K(K−1)/2<M≤K(K+1)/2, which is equivalent to $\left(-1+\sqrt{1+8M}\right)/2\leq K<\left(1+\sqrt{1+8M}\right)/2$. Hence, we can obtain *K* from a code-num *M* as

(1) $$K=\left[\frac{1+\sqrt{1+8M}}{2}\right],$$

where [*X]* returns the largest integer that is smaller than *X*.

Now, let us introduce *X*, which is given by

(2) $$X=M-\frac{K\left(K-1\right)}{2}.$$

With *K* and *X*, the binary code for each *M* is encoded as a bit stream composed of the MSB (=”1”) followed by *K* − (*X* − 1) zeros and *X* − 1 ones. For example, when *M* = 10, *K* and *X* are 1 and 4 according to Equations (1) and (2), respectively. Thus, the corresponding binary cluster is represented by “10111” as it has one (=4 − (4 − 1)) “0” bit and three (=4 − 1) “1” bits, which is identical to the binary cluster for M = 10 in Table 1.

The unary code is used for a prefix of the exponential Golomb code that is adopted for video compression codecs, e.g., H.264 [13]. In the unary code, a code-num *N* is encoded as *N*-1 “0”s followed by one “1”. For example, the unary codes for *M* = 1, 2, 3, and 4 are “1”, “01”, “001” and “0001”, respectively. The unary code is a simple and fast coding method, but its bit length increases with the integer value to be encoded. For the code-num 10, the unary code has a bit length of 10. However, the bit length of encoded code by the proposed method is 5 (i.e., 10111), yielding 50% compression efficiency in the prefix encoding. This efficiency becomes higher for a larger code-num. 

### 2.2. Proposed BL Code Encoding

In the exponential Golomb code, a binary code that directly converts a decimal to binary numbers with variable-width strings is concatenated as a suffix code. The exponential Golomb code using the unary code is an optimal code when integers follow a geometric distribution. Thus, it is useful for image/video compression. However, as the amplitude of the ultrasound signal follows the Rayleigh distribution [18], the efficiency of compression would be lower if the exponential Golomb code is used. To overcome this limitation, our BL code uses the proposed prefix and the binary coded decimal with increasing bit lengths as the suffix code as shown in Table 2. Note that the reversed binary cluster is used as a prefix to preserve the unique decodability. Otherwise, the same bit stream for different integer *Z* can be obtained with a non-reversed form. For example, assuming an encoded bit stream “101110”, the prefix of encoded code can be “101”, “1011”, and “10111” with a non-reversed form. However, if we use a reversed form, it can be readily found that the prefix is “101”. Table 2 lists some examples of the conventional Golomb and BL codes. In the proposed method, we can distinguish the prefix and the suffix parts with “01” (i.e., the reversed form of the seed binary cluster in Table 1). 

The BL code encodes an integer *Z* as follows. Empirically, we found that the code-num *M* of prefix can be obtained from a random integer *Z* as

(3) $$M=ceil\left(\log_{2}\left(\left(Z+2^{S}\right)/2^{S}\right)\right),$$

where *ceil*(·) is the ceiling function and *S* is an external integer parameter satisfying *S* ≥ 1 The number of bits allocated for the suffix code can be adjusted depending on *S*. In this study, we use the BL code with *S* = 1 for encoding. With *M* by Equation (3), *K* and *X* can be obtained from Equations (1) and (2), and then, the prefix for BL code is generated as described above in Section 2.1. Finally, the suffix of the proposed code, which uses the binary coded decimal (see Table 2), is obtained as:

(4) $$suffix=bin\left[Z-2^{S}\times \left(2^{M-1}-1\right)-1\right],$$

where *bin*[·] represents the decimal-to-binary converter. It is worth noting that from Equations (3) and (4), the bit length of the suffix of the proposed BL code is given by *M* + (*S −* 1).

**Figure 1:**
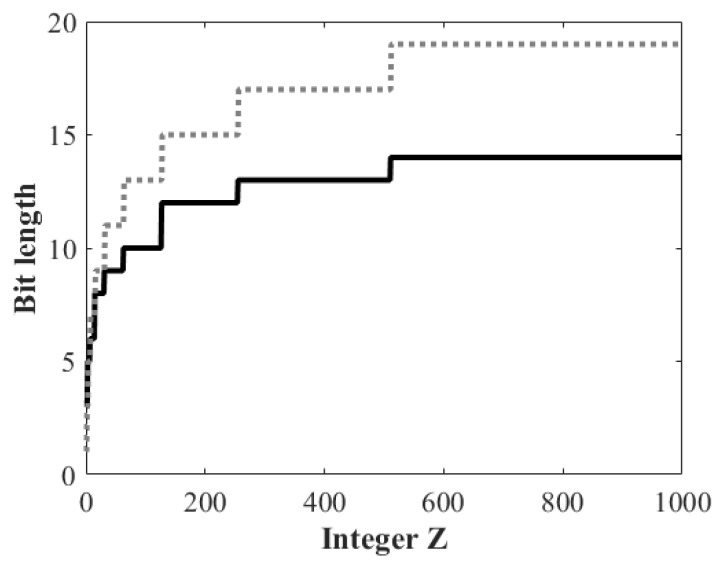
Comparison of bit lengths of encoded integers *Z* from 1 to 1000 by using the proposed and exponential Golomb code methods.

Figure 1 shows the bit lengths of encoded integers *Z* from 1 to 1000 by using the proposed and exponential Golomb code methods. Note that the bit lengths of encoded integers vary as a function of the integer, which lead to a reduced data rate compared to the fixed-width (e.g., 16 bits) binary coding. One can see that the proposed method achieves a higher compression ratio than the exponential Golomb code. This efficiency stems mainly from the proposed prefix encoding method described in Section 2.1. For example, the proposed method requires 10 bits and 14 bits for encoding two integers, 100 and 1000, respectively, while the exponential Golomb code encodes them into 13 bits and 19 bits, yielding 23.1% and 26.3% improved compression ratio, respectively.

### 2.3. BL Code Decoding

**Figure 2:**
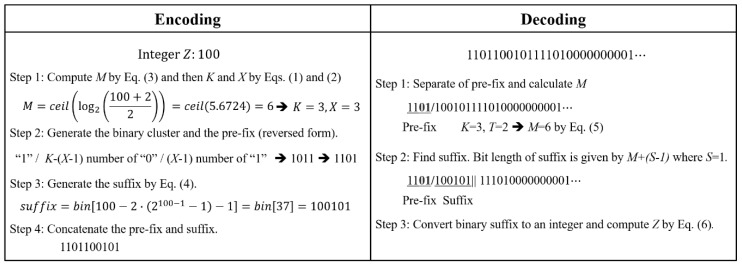
Encoding and decoding procedures of the proposed BL code.

The encoding and decoding procedures for the proposed BL code are shown in Figure 2. For decoding, we must first separate the prefix by finding “01” from the input bit stream (Step 1 in the decoding procedure in Figure 2). Then, from Equation (2), the corresponding code-num *M* is obtained as:

(5) $$M=\frac{K\left(K-1\right)}{2}+T+1,$$

where *K* is the group index, which is the bit length of the prefix excluding the MSB, and *T* is the number of continuous “1”s, i.e., *T* = *X* − 1, in the top (left) to bottom (right) bit direction in the prefix. For the prefix “1101” in Figure 2, *K* = 3 and *T* = 2, and hence *M* = 6 according to Equation (5). Then, the bit length of the corresponding suffix is six, as it is equal to *M* + (*S* − 1) where *S* = 1 in this study. Thus, the suffix is the 6-bit binary code “100101” following the prefix, of which the decimal value $suffix_{10}$ is 37. Finally, the integer *Z* is obtained from Equation (4) as:

(6) $$Z=suffix_{10}+2^{S}\times \left(2^{M-1}-1\right)+1.$$

From Equation (6), it can be readily computed that the integer *Z* for “1101100101” is 100. The rest of the bit stream, “111010000000001”, is decoded as 1024 using the same procedure described above.

## 3. Performance Evaluation and Discussion

**Figure 3:**
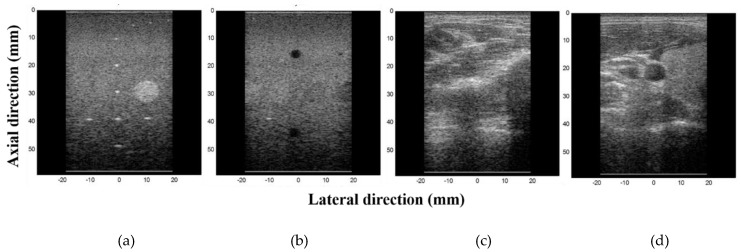
Ultrasound images showing (**a**) point targets, (**b**) cysts, (**c**) nerve and (**d**), which were constructed by utilizing the proposed BL code.

To evaluate the performance of the proposed method, we acquired two *in vitro* and two *in vivo* data sets using a portable ultrasound imaging system [9]. For each data set, we acquired pre-beamformed, beamformed and baseband inphase/quadrature (*I*/*Q*) after beamformation data by using an 8-MHz linear array with a sampling rate of 40 MHz. The ultrasound images constructed by using the four data sets are shown in Figure 3. In this study, we compute the compression ratio for the quantitative evaluation. The compression ratio is defined by

(7) $$\mathrm{Compression}\;\mathrm{ratio}\left(\%\right)=\left(1-\frac{\mathrm{Compressed}\;\mathrm{data}}{\mathrm{uncompressed}\;\mathrm{data}\;\mathrm{size}}\right)\times 100.$$

**Figure 4:**
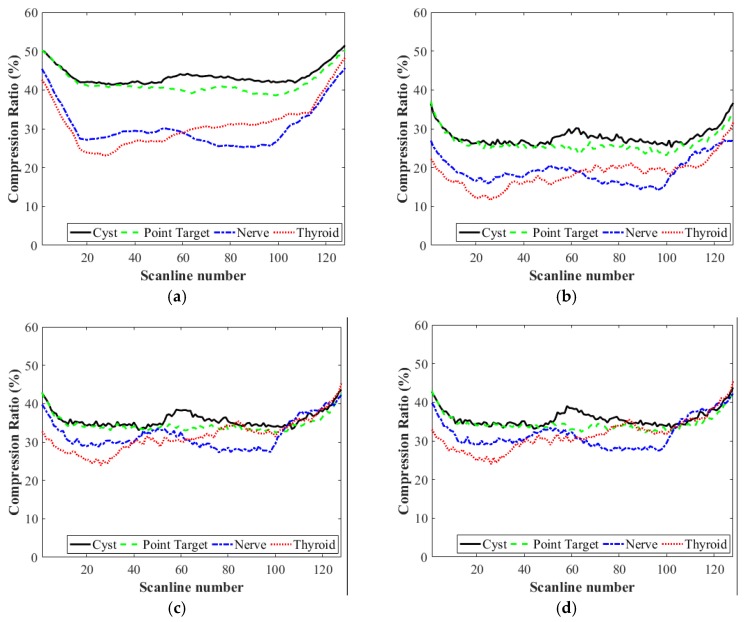
Compression ratios (%) of (**a**) pre-beamformed, (**b**) beamformed, (**c**) inphase (I), and (**d**) quadrature (Q) data that were used to construct the images of point targets, cysts, nerve, and thyroid in Figure 3.

Figure 4 shows the compression ratios of the pre-beamformed, beamformed and IQ data for each data set. The average compression ratios for the images of point targets, cysts, nerve and thyroid are, respectively, 41.2%, 43.6%, 30.6% and 31.2% with the pre-beamformed data and 26.2%, 27.9%, 19.2% and 18.5% with the beamformed data. *I* and *Q* data sets yield the same compression ratios; 34.6% (point targets), 35.9% (cysts), 31.9% (nerve) and 31.6% (thyroid). As shown in Figure 4, the *in vitro* data sets for the point target and cyst images in Figure 3a,b, respectively, provide higher compression ratios than the *in vivo* images in Figure 3c,d. *In vitro* images exhibit mostly speckles that appear as granular patterns and few strong reflectors (e.g., point targets and masses). The speckle patterns are produced by random interferences between coherent backscattered waves. Thus, the amplitudes of speckles are typically much lower than those from the strong reflectors [19]. For this reason, the *in vitro* data sets yield higher compression ratios, especially for the pre-beamformed RF data. By contrast, the baseband *I*/*Q* data produce similar compression ratios for each data set. Note that the compression ratios are identical for the *I* and *Q* data as the data have the same amplitude with a π/2 phase difference. As the envelope amplitude of the beamformed data is $\sqrt{I^{2}+Q^{2}}$, the amplitude of *I* (or *Q*) data is typically smaller than that of the beamformed data, resulting in a higher compression ratio than that from beamformed data.

**Figure 5:**
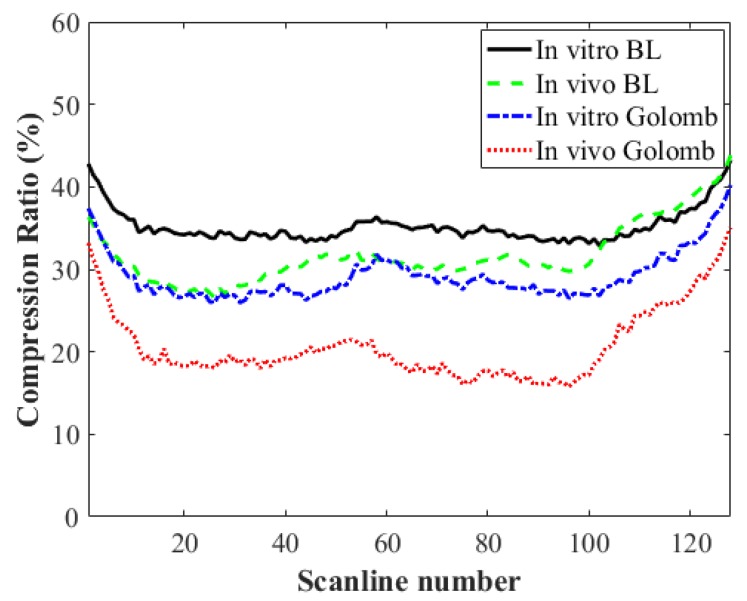
Comparison of compression ratios (%) of the proposed BL and exponential Golomb codes.

The comparison of compression ratios of the proposed BL and the conventional exponential Golomb codes is shown in Figure 5. The exponential Golomb code is used in H.264 image/video compression standard and is preferred over other lossless compression methods since it can be implemented with low computational load or hardware complexity. In addition, it provides a higher compression ratio on medical imaging data than other existing codes [20]. Thus, we compared the results obtained by the exponential Golomb and proposed BL codes. Here, we only compare the compression results for the baseband *I*/*Q* data sets as they provide similar compression ratios for both the *in vitro* and *in vivo* data sets. As shown in Figure 5, the proposed BL code yields higher compression ratios than the exponential Golomb code. The average compression ratios with the proposed BL and exponential Golomb codes were 35.2% (*in vitro* BL) and 29.1% (*in vitro* Golomb), respectively, for *in vitro* data sets, while the ratios decreased to 31.7% (*in vivo* BL) and 20.7% (*in vivo* Golomb), respectively, for *in vivo* data sets. From the experiments, the proposed method showed improved compression ratio compared to the exponential Golomb code. Previously, a compression method for functional magnetic resonance imaging (fMRI) data was developed [21]. Based on analysis of the probability distribution of fMRI data, they proposed a new compression method that assigns smaller binary codes to high values and original code for small value. Although the ultrasound signal follows the Rayleigh distribution and its shape varies depending on the tissue type, similar approach may improve the compression ratio of the proposed method.

The computational time of the exponential Golomb and proposed BL codes were tested using Microsoft Visual Studio 2017 on an Intel Core i5 CPU without any optimization technique. The average encoding times for the exponential Golomb and BL codes were, respectively, 0.52 and 0.67 s, and those were 5.65 and 4.89 s for decoding. If we employ a GPU for decoding, the processing time can be reduced. In addition, the processing time will be further reduced by using look-up table and other optimization techniques for real-time application, which is being conducted at this present.

Recently, ultrasound analog-front-end chips including programmable low noise amplifiers, gain amplifiers, LPFs, 12/14-bit ADCs, and quadrature demodulators with a decimator were introduced by some vendors such as TI and Analog Devices. In these chips, the demodulation is performed after ADC followed by the decimation with a factor of up to 16. For example, in 64-channel mid/low-end ultrasound imaging systems with a 12-bit ADC and sampling rate of 20 MHz, the data transfer rate can be reduced to 3.8 Gbits per second (Gbps) for the pre-beamformed *I* and *Q* data after digital demodulation and four-fold decimation by incorporating such chips. Note that the total data rate to transfer both *I* and *Q* data is 7.6 Gbps. This data rate does not meet the USB 3.0 specification. However, with the proposed method, the data rate can be further reduced by 30%; then, the data rate would be 2.6 Gbps for the *I*/*Q* data, lowering the total data rate to 5.2 Gbps, which could be handled by USB 3.0 devices marginally. 

## 4. Conclusions

In this paper, we proposed a lossless compression method, called BL code, for real-time software-based ultrasound imaging. The proposed method improved the compression ratio of the exponential Golomb code by reducing the prefix length. Because the bit length of the proposed prefix slowly increases as the code-num increases, an improved compression ratio was obtained. As the proposed method can encode integers using simple calculations, it can be implemented in real-time. The performance of the proposed method was validated using sample sets of ultrasound data. Our method could be applied in a wide range of data processing applications, especially in ultrasound image systems. In the future, we will implement the proposed method in an FPGA chip with a USB 3.0 interface on ultrasound imaging systems and assess its performance.

## Figures and Tables

**Table 1:** Binary cluster patterns from the proposed and unary code methods.

Code_Num (M)	Binary Cluster	Reversed Form of Binary Cluster (Prefix)	Group Index (K) (Bit Length of Binary Cluster-1)
1	10	01	1
2	100	001	2
3	101	101	2
4	1000	0001	3
5	1001	1001	3
6	1011	1101	3
7	10000	00001	4
8	10001	10001	4
9	10011	11001	4
10	10111	11101	4
…	…	…	…

**Table 2:** Encoded codes using the conventional golomb and BL codes.

Integer (Z)	Prefix (Unary Code)	Suffix	Exponential Golomb Code	Proposed Prefix	Suffix	BL Code
1	1	-	1	01	0	010
2	01	0	010	01	1	011
3	01	1	011	001	00	00100
4	001	00	00100	001	01	00101
5	001	01	00101	001	10	00110
6	001	10	00110	001	11	00111
7	001	11	00111	101	000	101000
8	0001	000	0001000	101	001	101001
9	0001	001	0001001	101	010	101010
10	0001	010	0001010	101	011	101011
11	0001	011	0001011	101	100	101100
12	0001	100	0001100	101	101	101101
13	0001	101	0001101	101	110	101110
14	0001	110	0001110	101	111	101111
15	0001	111	0001111	0001	0000	0001000
16	00001	0000	000010000	0001	0001	00010001
…	…	…	…	…	…	…

## Abbreviations

The following abbreviations are used in this manuscript:

ASIC	Application-Specific Integrated Circuit
FPGA	Field-Programmable Gate Array
GPU	Graphic Processing Unit
ADC	Analog-to-Digital Convertor
PCI Express	Peripheral Component Interconnect Express
USB	Universal Serial Bus
RF	Radio-Frequency
MPEG	Moving Picture Experts Group
MSB	Most Significant Bit
I/Q	Inphase/Quadrature
fMRI	Functional Magnetic Resonance Imaging
